# Adaptive changes induced by noble-metal nanostructures *in vitro* and *in vivo*

**DOI:** 10.7150/thno.42569

**Published:** 2020-04-27

**Authors:** Qianqian Huang, Jinchao Zhang, Yuanyuan Zhang, Peter Timashev, Xiaowei Ma, Xing-Jie Liang

**Affiliations:** 1 CAS Key Laboratory for Biomedical Effects of Nanomaterials and Nanosafety, CAS Center for Excellence in Nanoscience, National Center for Nanoscience and Technology of China, Beijing 100190, China; 2 University of Chinese Academy of Sciences, Beijing 100049, China; 3 Sino-Danish Center for Education and Research, Sino-Danish College of University of Chinese Academy of Sciences, Beijing, 100049, China; 4 Key Laboratory of Medicinal Chemistry and Molecular Diagnosis of the Ministry of Education, College of Chemistry & Environmental Science, Hebei University, Baoding 071002, China; 5 Wake Forest Institute for Regenerative Medicine, Wake Forest University School of Medicine, Winston-Salem, North Carolina, USA; 6 Institute for Regenerative Medicine, Sechenov University, Moscow, Russia

**Keywords:** noble-metal nanostructures (NMNs), applications, exposure, adaptive changes, *in vitro* and *in vivo*, proper implementation.

## Abstract

The unique features of noble-metal nanostructures (NMNs) are leading to unprecedented expansion of research and exploration of their application in therapeutics, diagnostics and bioimaging fields. With the ever-growing applications of NMNs, both therapeutic and environmental NMNs are likely to be exposed to tissues and organs, requiring careful studies towards their biological effects *in vitro* and *in vivo*. Upon NMNs exposure, tissues and cells may undergo a series of adaptive changes both in morphology and function. At the cellular level, the accumulation of NMNs in various subcellular organelles including lysosomes, endoplasmic reticulum, Golgi apparatus, mitochondria, and nucleus may interfere with their functions, causing changes in a variety of cellular functions, such as digestion, protein synthesis and secretion, energy metabolism, mitochondrial respiration, and proliferation. In animals, retention of NMNs in metabolic-, respiratory-, immune-related, and other organs can trigger significant physiological and pathological changes to these organs and influence their functions. Exploring how NMNs interact with tissues and cells and the underlying mechanisms are of vital importance for their future applications. Here, we illustrate the characteristics of NMNs-induced adaptive changes both *in vitro* and* in vivo*. Potential strategies in the design of NMNs are also discussed to take advantage of beneficial adaptive changes and avoid unfavorable changes for the proper implementation of these nanoplatforms.

## Introduction

The integration of nanoscience and biotechnology has spawned an increasing field of research which possesses immense potential to positively impact public health. In this amazing arena, the technological innovation of synthesizing materials at nanoscale level has provided great opportunities to achieve potential clinical applications encompassing healthcare treatment, diagnosis and imaging 1, 2. NMNs such as gold, silver and platinum are particularly attractive for theranostics due to their unique and promising features. Generally, noble metals refer to the metallic chemical elements in groups VIIb, VIII and 1b of the second and third transition series of the periodic table, including rhodium (Rh), ruthenium (Ru), palladium (Pd), silver (Ag), osmium (Os), iridium (Ir), platinum (Pt), and gold (Au), in order of atomic number 3. The most important characteristics of noble metals are their outstanding resistance to a wide range of corrosive attack and their stability even under conditions where base metals are oxidized rapidly. The history of noble metals dates back to as early as the Egyptian First Dynasty 4. They belong to a class of precious elements that have been found with a wide range of applications such as electronic devices, aerospace engineering, and most importantly, the health care system 3. Most NMNs are eclectic nontoxic and biocompatible agents. Table 1 shows a brief comparison of different types of NMNs and their main applications in academic studies. Among all NMNs, Au and Ag nanoparticles are the most extensively studied as they could be easily synthesized and are relatively safe. The intriguing physiochemical properties of NMNs such as plasmonic, optical, photothermal, and catalytic abilities allow them to act as efficient biosensing and imaging labels 5. By means of strong metal-thiolate bonds, they can be conveniently functionalized with biomolecules and exert multiple biological functions 6, 7. Furthermore, recent investigations have demonstrated broad application prospects of NMNs as self-therapeutic agents 8, 9.

The specific physicochemical properties of NMNs will result in increased activities within biological systems. With the increasing application of NMNs, both therapeutic and environmental NMNs are likely to be exposed to tissues and cells. Inert NMNs with excellent stability enable them to persist in complex biological systems for a long time. In general, NMNs that do not cause serious acute changes in cells or tissues are often considered to be biocompatible. How cells response to NMNs is of vital importance for their biological changes and survival. Tissue, organs and cells may undergo a series of adaptive changes upon NMNs exposure. Basic understanding of how NMNs interact with cells, organs, tissues and the underlying mechanisms are crucial for their various applications. Through in-depth study of these adaptive changes, we can skillfully take advantage of favorable adaptive changes and avoid unfavorable changes in order to obtain fantastic nanosystems 10. In this review, we give a summary of the characteristics of NMNs-induced adaptive changes and provide detailed understanding of these changes both *in vitro* and* in vivo*. Moreover, we discuss the potential usage of NMNs-induced beneficial adaptive changes for proper implementation of exquisite nanoplatforms.

## Characteristics of NMNs-induced adaptive changes *in vitro* and* in vivo*

NMNs can enter the body through inhalation, skin exposure, gastrointestinal absorption and biomedical applications. NMNs entering vascular system through various pathways will tend to be further distributed to peripheral tissues, organs, and cells, or be absorbed in the spleen and bone marrow 3. After reaching different tissues and organs, NMNs will interact with histocyte immediately and continuously. Cells will gradually evolve adaptive changes to achieve tolerance to NMNs and new cellular homeostasis, which is considered as basic condition for their survival in response to external stimulus and stressors.

The chemotherapy drug tolerance, which is described as the corresponding mechanisms evolved by cells to lose sensitivity to chemotherapeutics, is an example of cells gaining tolerance to improve survival 34. Similarly, with extensive exposure of cells to NMNs, a series of adaptive changes both* in vitro* and *in vivo* will been seen on cells for better survival. When subjected to repeated NMNs stimulation, cells may even lose sensitivity to NMNs, leading to reduced cellular accumulation and change of tolerance-related genes of NMNs. For example, *Priscila et al*. found a single administration of Au nanoparticles caused long-term genetic changes in cells, and these genes were involved in oxidative damage, cell cycle regulation, and inflammation 35.

Current research of biological effects of NMNs mainly focused on the short-term interaction between nanomaterials and biological systems and subsequent consequences. At the cellular level, researchers focused on the influences of NMNs on organelles, cell morphology, cell cycle, cell proliferation, and genes, etc. Most changes induced by NMNs are related to reactive oxygen species (ROS), known as oxidative stress. ROS possess multi-functions in cellular biology such as cellular proliferation, differentiation and death, with ROS production a key factor in NMNs-induced changes 36. NMNs-related stress is due to their smallness, notably large surface area per unit mass and high surface reactivity. When internalized into cells, NMNs will interact with intracellular nicotinamide adenine dinucleotide phosphate oxidase. The unique physicochemical reactivities of NMNs are correlated with the ability to trigger increased expression of pro-inflammatory and fibrotic cytokines and activation of inflammatory cells like macrophages and neutrophils. Cells show potent antioxidant defenses capable of overcoming oxidative stress and restoring redox balance when exposed to low concentrations of NMNs. By contrast, exposure to high concentrations of NMNs overwhelms cellular antioxidant systems and results in a sequence of pathological events including inflammation, fibrosis, genotoxicity, and carcinogenesis 37. The sizes of nanomaterials are comparable to those of viruses or bacteria for which our immune system has elaborated sophisticated responses. Specific rather than random oxidative reactions are elicited by cells to raise a protective response different from cell death mechanism when exposed to NMNs, which is critical to NMNs intracellular fate and effects 38. Cellular changes such as organelle functions, cell growth and apoptosis are often reported. Lysosomes and endosomes are demonstrated to have the most significant changes in cells since they are in direct contact with NMNs 39. In the absence of membrane-penetrating peptides, internalized NMNs mainly accumulate in lysosomes without entering the cytosol. Nevertheless, with the pursuit of precision medicine, subcellular targeting has become one of the biggest breakthroughs in achieving optimum therapeutic effect with minimal dosing. With the assistance of organelle-targeted molecules, internalized NMNs will eventually find their way to specific organelles and subcellular structures 40. Lysosomes, endoplasmic reticulum (ER), Golgi apparatus, mitochondria, and nucleus are the primary targets for various therapeutics as their functions are intimately bounded up with cell proliferation, cell growth, differentiation, and cell death 41. The accumulation of NMNs in these core organelles will interfere with their functions and cause changes in a variety of cellular functions including cellular digestion, protein synthesis and secretion, cellular respiration, and cellular replication. To maintain the homeostasis of cellular degradation system, cells will synthesis more lysosomes to compensate the missed degradative function after lysosome swelling. The alternation in protein processing allows cells to be adaptive to nanomaterials stimulation by activating specific signaling pathway, secreting various chemical substances, and regulating functional protein synthesis. Even when intercellular oxygen is sufficient, cells may initiate glycolysis to satisfy their energy demand. In animals, there are heightened interest in the physiological and pathological effects of NMNs on tissues and organs. Superior biocompatibility and immune-compatibility are the main points of *in vivo* evaluation of nanomaterials. Scientists are concerned with the distribution of NMNs in tissues and organs and their biocompatibility, which is related to their safe applications. A plethora of studies have shown accumulation of NMNs in liver, kidney, lung, heart, spleen, and brain 42. Although they have various exposure pathways, NMNs are mainly stored in reticular endothelial system through first-pass metabolism. Due to their inertness and good biocompatibility, NMNs can withstand various influences of the body and remain relatively stable in tissues and organs. Internalized NMNs may store in these tissues and organs at different time points, resulting in unforeseen physiological and pathological effects on metabolism, respiration, consciousness and immunity.

With the widespread application of NMNs, both therapeutic and environmental NMNs are likely to be exposed to tissues, organs and cells for a long time. Research on long-term exposure of NMNs, although relatively rare, has been proved to be of vital importance to study adaptive changes induced by NMNs 43. When exposed to a certain amount of NMNs for a long time, cells may undergo a series of morphological and functional changes. Cellular response to NMNs is an attempt to try to ensure their survival. The adaptive changes caused by long-term NMNs exposure are different from short-term exposure, and long-term exposure causes relatively less cytotoxicity. *Kristen et al.* implemented a chronic *in vitro* model treated with low dosage of Ag nanoparticles. Chronic exposure at low dosages of Ag nanoparticles did not cause cytotoxic changes, but instead activated sustained stress and signaling responses. Long-term Ag nanoparticles treated cells operated normally with augmented stress response and improved cellular function compared to acutely treated cells, indicating that cells gradually adapted to long-term stimulation of NMNs 43. Similar to chemotherapeutic drug tolerance, cells become resistance to NMNs under long-term stimulation, thus losing sensitivity and inhibiting uptake of NMNs. Continuous exposure of Au nanoparticles at low doses results in rapid intracellular accumulation and increased ER stress. However, when the concentration of Au nanoparticles exceeds the intracellular concentration threshold, further uptake of Au nanoparticles is inhibited, thereby reducing ER stress 44. Chronic and low dosage of NMNs exposure may lead to prolonged changes in cell physiology, which are direct indicators of cellular responses to long-term stimulus. Acute Au nanoparticles exposed cells showed significant cell area decrease, but after long-term incubation, the decreased cell area recovered 35. All these results suggest that cells turn to change under both acute and chronic incubation of NMNs, and long-term exposed cells increase their adaptability to the stimulation for better survival.

## NMN-induced changes in cell morphology and functions

### Cellular morphology change by NMNs exposure

Cellular and subcellular morphology are responsible for intercellular communication, cell homeostasis and functions. Recent advances in high-resolution imaging and biophysical technology have made it feasible to directly evaluate detailed changes of cellular and subcellular morphology in response to NMNs exposure 45. When in contact with cells, nanomaterials are typically ingested by endocytosis, and are trapped inside endosomes or lysosomes without entering cytoplasm 46. Once entering the cells, NMNs would have profound effects on cellular structures and morphologically related functions. The alterations of cytoskeleton and organelle structure caused by NMNs exposure have been reported in multiple studies.

**Figure 1:**
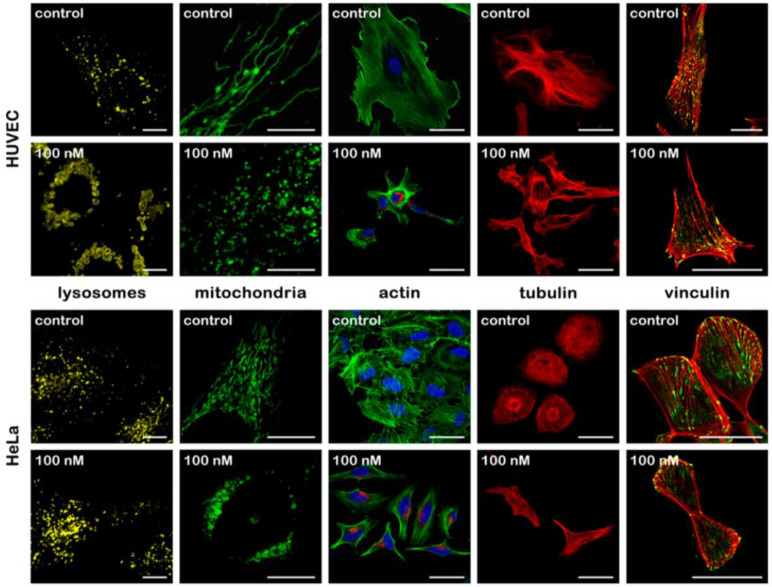
Morphological changes of different cellular structures without and after Au nanoparticles treatment in HUVECs and HeLa cells. Reproduced with permission from ref 54. Copyright 2017 American Chemical Society.

Cell cytoskeleton is the center part for maintaining cell morphology. *Pernodet et al.* found that the presence of Au nanoparticles affected cell morphology in a concentration-dependent manner. Upon Au nanoparticles exposure, multi parameters of actin stress fibers in human dermal fibroblasts, such as diameter, density, stretching state changed, consequently affecting cell shape, growth and viability 47. A similar study demonstrated concentration-dependent effects of Au nanoparticles on actin and tubulin. Cellular morphology change was observed at concentrations above 50nM 48. Additional research focused on the relationship between nanoparticles morphology change and alternation of cytoskeleton. Different sized nanoparticles induced different extent of cytoskeletal filament disruption 49. Another study about the effects of nanoparticles modification on cellular uptake and cytotoxicity showed that naked nanoparticles caused changes in cell morphology, while high concentrations of PEGylated Au spheres did not cause such changes in cells 50. The aggregation states of Au nanoparticles also cause various cellular responses in cell cytoskeleton. After uptake of aggregated and non-aggregated cationic Au nanoparticles, human dermal fibroblast cells exhibited varying degrees of F-actin fiber disruption with the appearance of actin dots 51. Studies on the influence of NMN on cell morphology are mainly focused on short-term effect. Some new findings also demonstrated the impact of acute Ag nanoparticles treatment on cellular membrane and cytoskeleton reconstruction 52, 53. Recently, small but significant morphology change was found in cells at each tested time point under long time exposure (20 weeks) of Au nanoparticles. For all nonchronic nanoparticles-exposed cells, cell area decreased significantly, which reflected the contraction of cytoskeleton, while after long-term culture, the decrease in cell area recovered 35. Our group further checked the alternation of cellular and subcellular morphology induced by NMNs (Figure 1). Cells undergo organelles structures change, alterations in cytoskeleton and reduction of focal adhesion contact area after Au nanoparticles treatment. The effects of NMNs on cellular morphology can be observed at concentrations lower than that of on cell viability, giving an important quantitative indicator to evaluate the effects of NMNs 54.

The impact of NMNs on cytoskeleton may influence the behaviors associated with cytoskeleton reconstruction, including cell junction, division and movement, since the components of cytoskeleton are known for their roles in these behaviors 55. *Lin et al.* observed the intercellular tight junctions were loosed by different sized Au nanoparticles 56. Our previous work demonstrated the focal adhesion contact area and the number of filopodia were reduced after Au nanoparticles treatment 54. The disruption of tight junction by NMNs was also observed by *Xu et al*. They demonstrated that Ag nanoparticles could induce destruction of tight junctions and cross through the blood brain barrier (BBB) 57. Furthermore, cell division is subject to change, as well as cell migration. *Lee et al*. mentioned the regulation of cytoskeletal structure by Au nanoparticles repressed cell division. This could be further explained by the transformation of crosstalk between actin and β-tubulins after nanoparticles incubation 58. *Rafailovich et al*. analyzed cellular effect of Au nanoparticles on human adipose-derived stromal cells. Cells exhibited concentration-dependent increase in population doubling times, decrease in cell motility, and contraction of cell-mediated collagen following Au nanoparticles exposure 59.

It is clear that the uptake of NMNs has great impacts on cellular morphology. Cytoskeleton plays an important role on attaching and arranging organelles inside cells. The effect of NMNs on cytoskeleton in turn affects organelles that bind to it. cellular and subcellular morphology changes occur not just due to direct aggregation of NMNs inside cells. Complex cellular network lets us take the indirect interactions among different intracellular organelles into account. Under the treatment of NMNs, cells turn to change their morphology followed by altering morphologically related cellular functions, which increase their adaptability to NMNs stimulation.

### Alteration of core organelles-related cell functions by NMNs exposure

**Figure 2:**
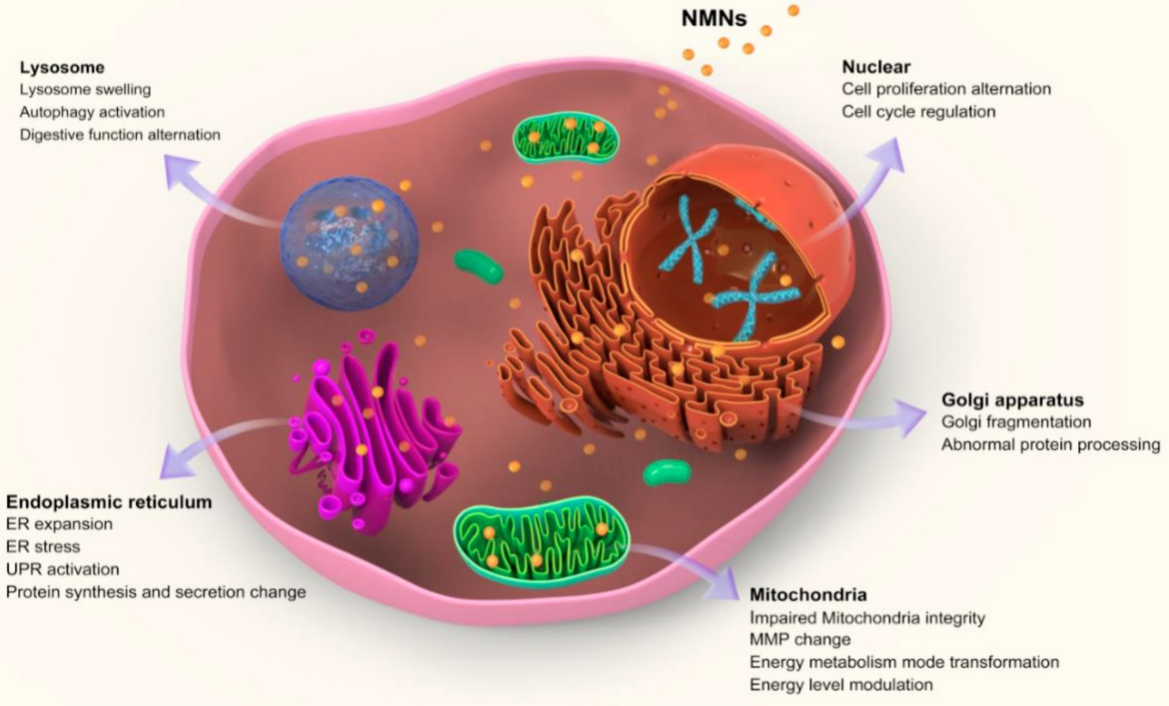
Schematic illustrating alterations of core sub-organelles and related cell functions by NMNs exposure.

After entering cells, nanoparticles firstly stay inside acidic lysosomes, or with the assist of organelle targeting agents, nanoparticles will then accumulate in targeted organelles like mitochondria, ER, nucleus, etc. 60. Upon NMNs exposure, organelle morphologies are altered either through direct nanoparticles accumulation or indirect subcellular interaction. Moreover, the adaptive changes of subcellular morphologies induced by NMNs will result in associated adjustment of cell behaviors and organelle-related cell functions. These alternations make cells adaptable in response to environmental changes (Figure 2).

#### Cellular digestion

Nanoparticles are ingested by cells mainly through endocytic pathway and will stay inside acidic lysosomes firstly. Our previous work found Au nanoparticles were ingested by cells in a size-dependent manner. Large number of Au nanoparticles accumulated in lysosomes led to lysosomes enlargement followed by lysosome alkalization, which affected autophagy 61. Remarkable lysosome swelling due to nanoparticles accumulation was also identified by biological transmission electron microscope 62. Lysosomes is crucial for maintaining cell homeostasis and is considered to be the center of sophisticated signal transmission and metabolism 63. It the end point of endocytic pathway and the digestive organelle of both intracellular and exogenous cargos. Superior accumulation of NMNs in lysosomes greatly influences cellular digestion 39.

Autophagy is a lysosome-mediated degradative process to achieve the metabolic needs of cells and the renewal of certain organelles. During autophagy, self-cytoplasmic proteins or organelles are coated into vesicles and fused with lysosomes to form autolysosomes, then the engulfed contents will be degraded 64. There has been increasing interest in autophagy since it was first discovered in 1963. Numerous studies have reported that various NMNs are capable of inducing autophagy. *Zhang et al*. proposed that autophagy is a common cellular response induced by NMNs. HeLa cells treated with Pd nanoparticles at low concentrations induced autophagy through mTOR signaling pathway in a size-dependent manner. With increased particle size of incubated Pd nanoparticles, the lysosome degradation capability decreased 65. As evidenced by transmission electron microscopy, Ag nanoparticles activated autophagy and induced autophagosome formation 66. Au nanomaterials are widely used in biomedical field due to their low toxicity and good biocompatibility. Au nanoparticles promoted the expression of autophagic proteins and proved to be a novel kind of autophagy activator 62, 67. Our group further explored the underlying mechanism. Different sized Au nanoparticles entered cells in a size dependent manner and eventually accumulated in acid lysosomes. Accumulation of Au nanoparticles in lysosomes leads to lysosomal alkalization. By monitoring the degradation of autophagy substrate p62, we provided that the accumulation of autophagosome after Au nanoparticles exposure was owing to the blockade of autophagic flux rather than autophagy induction 61. Similarly, another work based on Ag nanoparticles engulfed by A549 cells also showed autophagosome accumulation was caused by autophagy flux blockade via lysosomal alkalization 68.

The aforementioned results give us detailed information about NMNs induced autophagy. The activation of autophagy is a cellular stress response to intracellular foreign bodies, which is essential for cellular self-protection, as well as the recycle of damaged proteins and organelles to sustain metabolism homeostasis 69. The presence of nanomaterials inflicts lysosomes alkalization, which causes partial loss of endolysosome function. In order to accommodate these changes, cells will synthesis more lysosomes and initiate autophagy to make up the missed degradative function. The induced autophagy thus is considered as a protective mechanism against NMNs stimulation 70. *Li et al*. analyzed the role of Au nanoparticles in inducing autophagy both *in vitro* and *in vivo*. Ag nanoparticles treatment induced autophagy via calmodulin-dependent protein kinase kinase β and adenosine 5′-monophosphate-activated protein kinase pathway. Inhibition of autophagy significantly enhanced cytotoxicity of Ag nanoparticles, suggesting autophagy played a protective role in Ag nanoparticles-induced cell apoptosis 71. Moreover, a key step in cell digestion is to take up the digested material into cells. The exposure of NMNs can influence micropinocytosis and thus may inhibit cell digestion. Long-time exposure of Au nanoparticles above intracellular concentration threshold inhibited micropinocytosis mechanism to cease further nanoparticles uptake, which adaptively reduced ER stress 44.

#### Protein synthesis and secretion

ER is the largest organelle in cells. ER and Golgi apparatus are responsible for membrane interaction and protein synthesis. They are central for regulating various signaling pathways and cell response to stress 72. With the pursuit of precision therapy, accumulating efforts have been made by researchers to target ER and Golgi apparatus with nanotechnology 73. *Xu et al.* observed ER expansion in astrocytes after Ag nanoparticles incubation 57. As the major organelle for protein folding, ER complete the folding process of incompletely folded proteins with the help of molecular chaperones and folding enzymes 74. NMNs exposure may impede protein folding capacity and thus lead to the accumulation of misfolded proteins in ER, named ER stress. It was shown that PEGylated nanogels containing Au nanoparticles increased the expression of ER stress-related proteins 75. With the accumulation of misfolded proteins in ER, the equilibrium between ER protein load and folding capacity under normal conditions will be damaged. In order to restore ER homeostasis to better withstand nanoparticles stimulation, an adaptive signaling pathway termed as unfolded protein response (UPR) will be activated, which result in protein synthesis and secretion changes 76. For example, Ag nanoparticles treatment led to accumulation and aggregation of misfolded proteins in cells which caused ER stress and activated UPR. Through this process, downregulation of Mcl-1 and xIAP protein expression as well as a processing of PARP protein were observed 77.

Golgi apparatus is the organelle for final processing and packaging of proteins. Vesicles from ER fuse with Golgi membrane and deliver the contents into Golgi cavity where newly synthesized proteins continue to be modified and packaged 78. Due to structural and functional continuity of ER and Golgi apparatus, NMNs-induced ER stress can also cause Golgi apparatus stress by affecting normal protein transportation. Alternation of Golgi apparatus by NMNs will ultimately affect protein transport and secretion. Recently, we first discovered Au nanoparticles increased cytoplasmic calcium and induced Golgi fragmentation, thereby impaired normal Golgi functions, while cell viability was not compromised. The fragmentation of Golgi apparatus induced by Au nanoparticles hampered normal Golgi functions, which led to abnormal protein processing and cellular adhesion change 79.

#### Energy level and cell fate

##### Mitochondrial respiration modulation by NMNs

Mitochondria are best known to be the powerhouse of cells, which are in charge of many important cellular functions. On one hand, mitochondria decompose carbohydrates to produce ATP through electron transport and oxidative phosphorylation, providing most of the energy needed for metabolism and movement of organisms. On the other hand, mitochondria are involved in various metabolic pathways and apoptosis 80. Hence, mitochondria determine cellular energy level and cellular fate and are attractive targets to treat mitochondria-associated diseases 73. Multiple studies have revealed NMNs-induced mitochondria morphology change. For example, *Pan et al*. reported Au nanoparticles with diameter of 1.4 nm compromised mitochondrial integrity, potential, and its substrate reduction 81. Localization of ammonium-modified Au nanoparticles in cellular mitochondria caused mitochondria swelling and enlargement 82. *Xu et al*. observed severe mitochondrial shrinkage after Ag nanoparticles treatment 57. Mitochondria are sensitive to external stimuli and are key regulators of cell survival and death 83. Previous research demonstrated that changes in mitochondria occurred at the lowest Au nanoparticles concentration compared with that of other organelles 54. When NMNs enter the cell, whether through direct mitochondrial aggregation or indirect interaction with the membrane, nanoparticles would trigger mitochondrial oxidative stress, affect mitochondrial respiration, and change cellular energy levels.

Mitochondria are more complex compared with other organelles because of the double-layer membranes. Most of ATP used by cells is synthesized by mitochondrial respiration via oxidative phosphorylation. This process causes a potential difference between mitochondrial inner and outer membranes. Although the double-layer membranes block many substances, harmful or therapeutic, from entering into mitochondria, mitochondrial membrane potential (MMP) makes it possible to transport bioactive molecules into mitochondria with assist of nanomaterials 41. A growing number of reports have revealed the modulation of mitochondrial respiration by NMNs, among which the most widely reported are changes in MMP. *Chen et al*. compared the effect of Au nanorods on cancer and normal cells. Selective accumulation and long-term retention of Au nanorods in cancer cell mitochondria led to destruction of MMP and increase of ROS. This may be used for mitochondria-targeted cancer therapy 84. They also analyzed the bioeffect of Ag nanoparticles. Electron transfer impairment in mitochondria was found after Ag nanoparticles incubation in their study, as well as impairment of ATP synthesis, which triggered oxidative stress 85. In addition to *in vitro* studies, *in vivo* analysis also revealed mitochondrial alteration after NMNs exposure. Prolonged exposure to a low dose of Ag nanoparticles was sufficient to cause alteration in hepatic mitochondrial function 86. Since the significant change of MMP after NMNs exposure, a chapter in *nanotoxicity* proposed an overview of nanomaterial-induced loss of MMP and presented a detailed method to accurately quantify the loss of MMP upon nanomaterials treatment 87.

**Figure 3:**
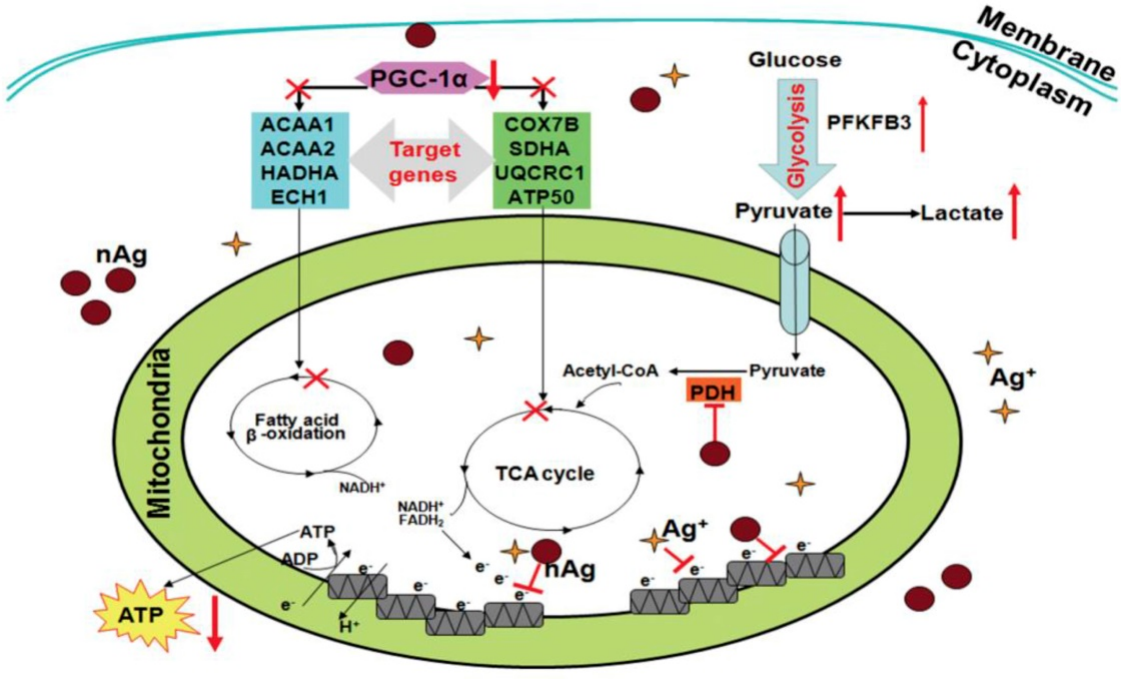
Schematic diagram showing the molecular bases underlying Ag nanoparticles-conducted reprograming of energy metabolism under sublethal exposure. Reproduced with permission from ref 89. Copyright 2014 American Chemical Society.

Under normal conditions, energy homeostasis is maintained within cells through concerted signaling and metabolic pathways. Mitochondria are the main organelle responsible for energy homeostasis in mammalian cells. Alternation of mitochondrial integrity or mitochondrial dysfunction may lead to severe impairments of energy metabolism, causing associated diseases. Therefore, the influences of NMNs on mitochondria require cells and organisms to maintain internal energy homeostasis to accommodate NMNs stimuli. Transformation between different energy metabolism modes is a cellular adaptive change to satisfy the energy demand for cell survival. For example, energy in normal cells is primarily produced by the metabolism of oxidative phosphorylation of reactive mitochondrial respiration, whereas in tumor cells, tumor cells initiate glycolysis-based metabolism for energy production simultaneously even in the presence of high oxygen concentration 88. When cells are exposed to NMNs, cells tend to shift energy metabolism mode to satisfy energy demand for their survival. *Chen et al*. studied Ag nanoparticles-mediated effects on energy metabolism in cells under sublethal exposure. Ag nanoparticles treatment at nontoxic concentrations resulted in compromised MMP and electron transfer along respiratory chain. Decline of ATP synthesis and attenuation of respiratory chain function led to initiation of glycolysis to compensate ATP decrease and satisfy basal energy demand for cell survival 89. As illustrated in Figure 3, this schematic diagram deciphers the mechanisms by which Ag nanoparticles incur an adaptive change of energy metabolism mode to glycolysis under sublethal exposure in both tumor and normal cells. Alterations of several energy metabolism related genes are involved. All these changes force cells to reprogram energy metabolism by switching from oxidative phosphorylation-based aerobic metabolism to anaerobic glycolysis. This adaption process makes up the energy decrease in response to NMNs exposure to satisfy energy demand for cell survival.

##### Nucleus change: cell proliferation and growth

Nucleus contains the vast majority of genetic material of cells and controls gene expression. It is surrounded by a bilayered nucleus membrane that separates genome from cytoplasm to ensure accurate DNA replication and gene expression. There are thousands of protein machines, namely nucleus pore complexes (NPCs), on nucleus to maintain material exchange and information transmission between nucleus and cytoplasm 90. Although the small-sized and highly selective NPCs are complicated biological barriers for exogenously applied macromolecules, some ultrasmall metal nanoparticles might still diffuse into nucleus 91. In addition, with the aid of nucleus-targeting ligands, various surface modifications of NMNs allow them to be accurately delivered to nucleus 40, 92. As the most important organelle of eukaryotic cells, nucleus performs central regulation function of cell proliferation, metabolism, and cell cycle. There is heightened interest in the influence of NMNs on nucleus since it is critical for cell survival. The direct and indirect interactions between NMNs and nucleus have significance effects on cellular nucleus, which may result in cell growth, proliferation, mitosis, and apoptosis alternation.

Among all kinds of NMNs, Au nanoparticles are most widely used for nucleus targeting due to their controllable ultrasmall size and tunable surface modification. In the research of *Mostafa et al.*, Au nanoparticles localized at nucleus broke DNA double-strand and induced cell cycle arrest in cancer cells. Nucleus change upon acute Au nanoparticles exposure led to the failure of complete cell mitosis 93. The size, shape, and even surface modification of Au nanoparticles may change multiple nucleus parameters significantly. Small Au nanospheres and nanoflowers altered nucleus morphology, organization and function of MCF-7 cells, but big Au nanospheres did not. The changes of nucleus organization and function greatly influenced cell survival 94. Another research focused on the interactions between cells and Au nanoparticles modified with different PEG layer properties. Au nanoparticles with all PEG grafting densities caused significant nucleus damage of cancer cells. The alternation of nucleus led to cell cycle arrest without apoptosis 95. The long-term effect of Au nanoparticles on nucleus also demonstrated modulation of cell cycle, and acute NMNs exposure was more harmful to cells compared with chronic treatment 35. In mouse embryonic fibroblasts, after treatment with low doses of cobalt nanoparticles for three months, in addition to changes in cell morphology, characteristics similar to tumor cells such as increased secretion of metalloproteinases, enhanced anchorage-independent cell proliferation were observed 96. NMNs and NMNs-induced oxidative stress will also influence nucleus components such as nuclear lamins and histones. *Zhao et al.* reported that Ag nanoparticles induced phosphorylation of histone H2AX followed by DNA double-strand breaks, which was synergistically enhanced by ultraviolet A exposure 97. *Jennifer et al.* indicated that Ag nanoparticles treatment reduced nucleus lamin B1, which resulted in the up-regulation of telomeric repeat binding factor protein expression and maintenance of telomere length 98. Stress-induced premature senescence (SIPS) or stress-associated premature senescence (SAPS) is a type of senescence appears after exposure of cells to stress by chemical, physical or biological agents, such as oxidants, ultraviolet radiation, and viruses, among which nanomaterials exposure is a typical example 99. After NMNs stimulation, chronic or acute, stressed cells developed a “senescence-like” phenotype. *Jennifer et al.* demonstrated for the first time that nanoparticles may stimulate SIPS. P53 and p21 levels were elevated and SIPS was observed in cell treated with Ag nanoparticles, suggesting that nanoparticles promote a telomere-focused cell adaptive response 98.

Under the stimulation of NMNs, cells not only undergo changes in nucleus morphology, but also in nucleus function and related cell behaviors. Cellular response to NMNs is an attempt by cells to ensure survival. The observed effects of NMNs on nucleus indicate that cells will become more like cancer cells to accommodate NMNs stimulation, such as enhanced cell proliferation and cell viability. These characters enable cells to better cope with damage caused by NMNs. Moreover, chronic treatment of NMNs may lead to prolonged changes in cell physiology. In other words, cells can adapt to long-term NMNs exposure, which can be considered as part of adaptive responses to long-term effects of external stimulus 35. However, when NMN-induced nucleus change is beyond their self-repair, cell death will appear 100.

##### Genetic alteration: DNA structure and gene expression

Genetic alteration testing is of vital importance in the safety assessment of NNMs as it may have implications to induce chronic diseases such as cardiovascular disease and mental disorders, or even cancer. The impact of NMNs on genes is affected by NMNs properties, such as the composition, size, shape, surface properties, and also affected by exposure time, cell type, and treatment regime 101. Genetic assessments of different NMNs have been performed, including their impacts on DNA structure, chromosome, gene expression, potential mutagenesis. Confocal microscopy images showed that localization of Au nanoparticles at nucleus of cancer cells broke DNA double-strand structure, which resulted in binucleate cell formation after mitosis 102. *Li et al.* identified the interaction energy between different types of nanoparticles and DNA. NMNs with high binding affinity to DNA like Ag and Au nanoparticles had greater potential to interfere with DNA replication 103. Chromosome abnormalities are a direct consequence of DNA structure changes. NMNs-induced DNA structure damage such as double strand breaks and misrepair of strand breaks will influence chromosome morphology, resulting in chromosome rearrangement 104. However, in the research of *Xia et al.*, although significant DNA breaks were observed after Au nanoparticles treatment, there was no significant difference in the incidence of chromosomal aberrations between cells treated with/without Au nanoparticles, indicating that cells were trying to repair DNA damage during this process 105. The impacts of NMNs on genetic structure will appear as changes in protein and gene expression. It is therefore not surprising to find up-regulation of genes associated with DNA repair after NMNs exposure 106. Many other genes associated with cell cycle 81, anti-apoptotic 104, stress response 107 also changed in various cell lines after NMNs exposure.

**Figure 4:**
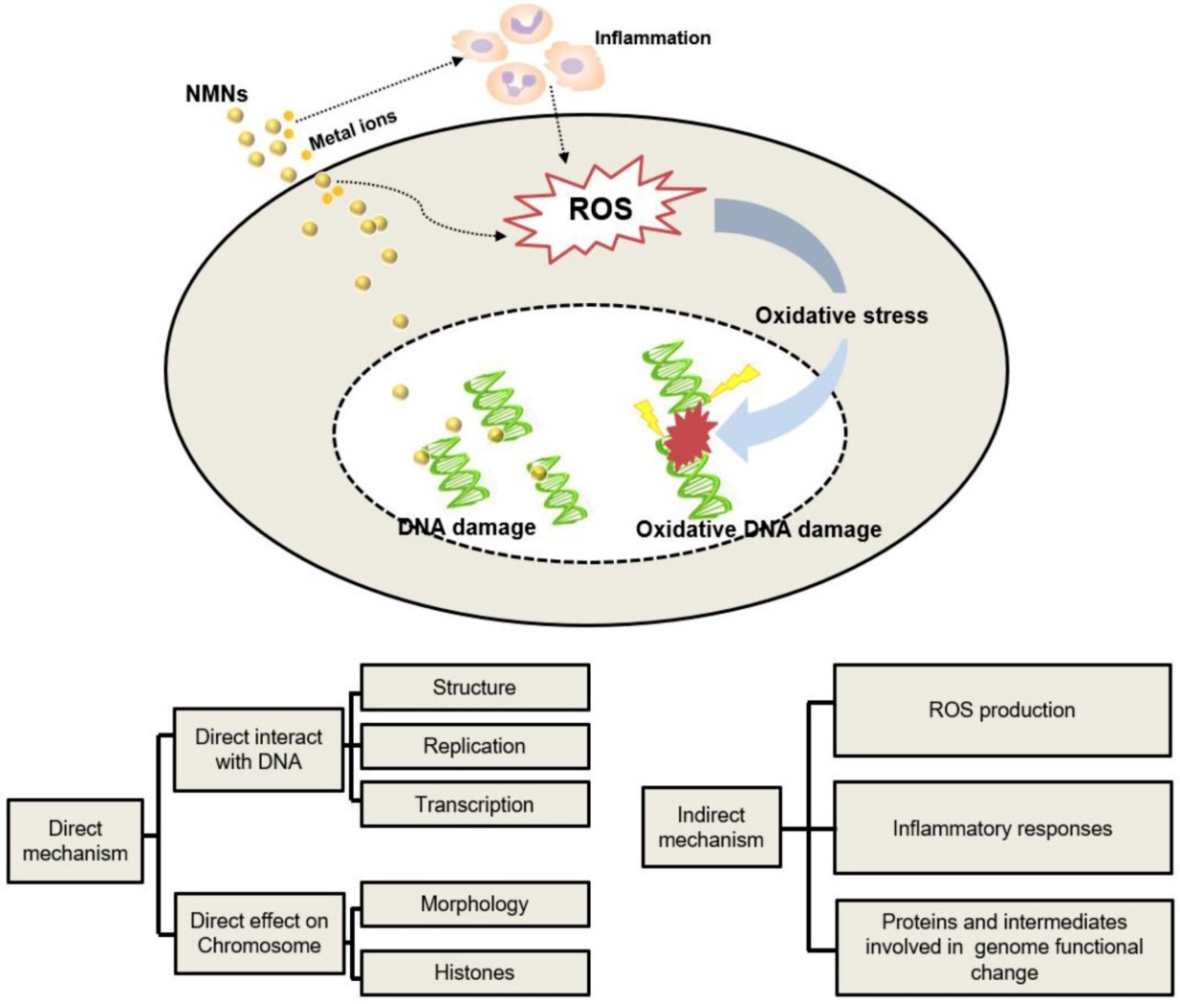
The mechanisms of NMNs-induced genetic alternation.

Published data indicate that there are direct and indirect mechanisms of NMNs-induced genetic alterations as summarized in Figure 4. Direct genetic changes in NMNs-treated cells occur as direct interactions of NMNs or their released ions with either nuclear DNA or mitochondrial DNA. The indirect mechanisms are that NMNs indirectly affect the molecules and intermediates involved in normal genome function or cell division. ROS production is considered to be the key factor causing NMNs-induced gene alternation 101. Production of ROS can cause DNA injury as well as changes in lipids, proteins, and other cellular components. Induced epigenetic alterations mediate chromosomal instability or locus specific gene expression changes, which possibly affect multiple cellular pathways and targets. Another mechanism of NNM-induced genetic alternation is mediated by extracellular ROS accumulation caused by inflammatory response. Activation of inflammatory responses of macrophages and neutrophils leads to production of ROS, which can also induce genetic alternations in nearby cells 108. In addition, some other molecular mechanisms have emerged recently that could explain the genetic changes caused by NMNs. For example, when NMNs accumulated inside endosomes/lysosomes abundantly, acidic conditions of endosomes/lysosomes will change accordingly and promote the release of metal ions or induce cascading intracellular gene toxicity 109. In these cases, cells initiate various responses to repair DNA damage and strive to adapt the stimulation of NMNs. Nevertheless, when NMNs-induced genetic alterations are beyond their repair capability or intracellular antioxidants are depleted, cells may undergo cell death via apoptosis or necrosis 104.

## Physiological and pathological effects of NMNs on tissues and organs

**Figure 5:**
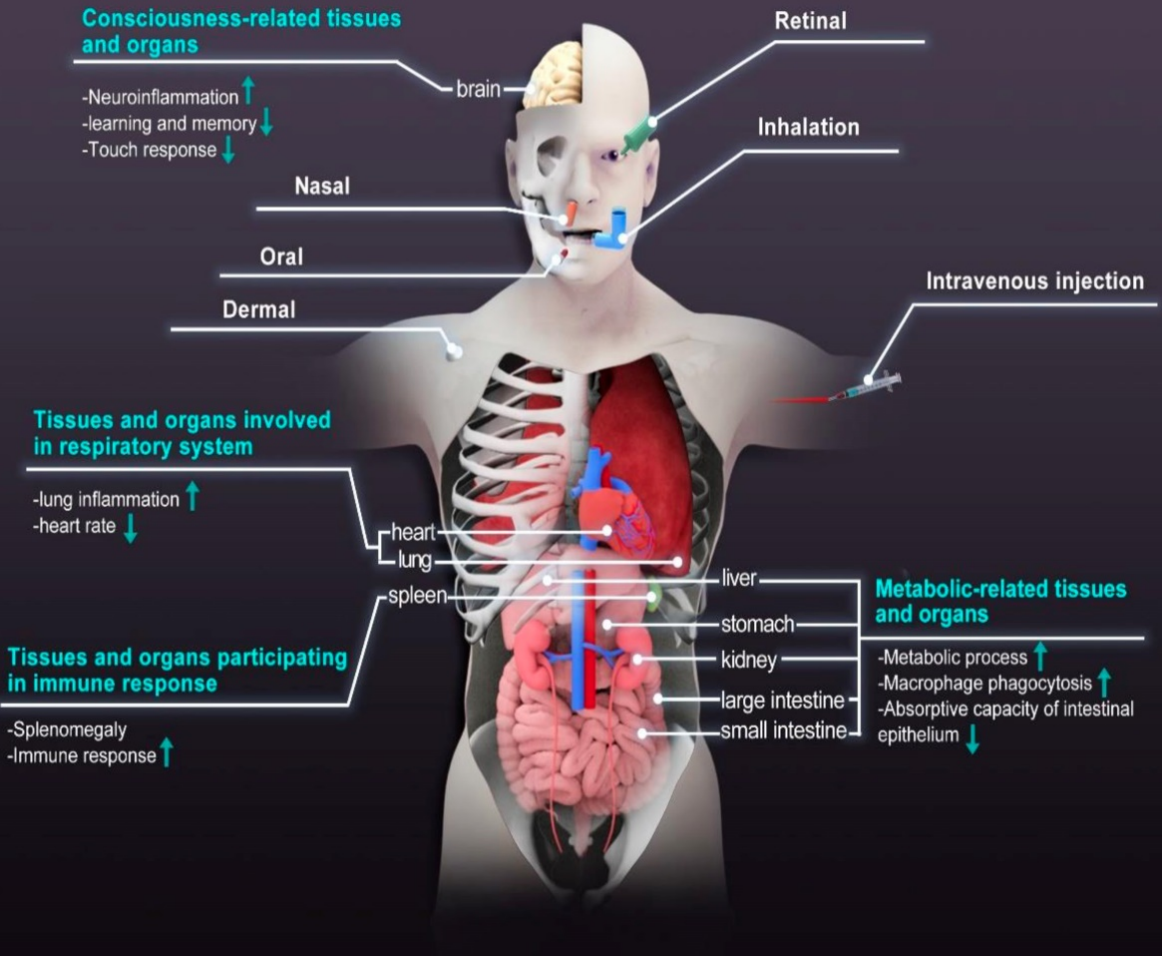
Routes of exposure commonly used for NMNs administration and physiological and pathological effects of NMNs on core tissues and organs.

The diverse applications of NMNs have led to increasing attention about their possible impact on human beings and the environment. Human beings are easily exposed to NMNs via ingestion, inhalation, and skin contact. Lately, the emergence of therapeutic drugs based on NMNs makes us more concerned about their fate after administration 42. More and more imaging modalities for quick and reliable mapping of NMNs distribution *in vivo* have been developed to optimize their physicochemical properties for various theranostic applications 110. Reports suggest that typical *in vivo* fate of NMNs depends on multi parameters such as size, shape, charge. These materials tend to accumulate within organs and are slowly degraded or excreted from the body 111. Due to non-biodegradable nature and prolonged circulation time, NMNs have high probability of accumulating in human body and interacting with tissues and organs. The long-time retention of NMNs in human body will have adverse effects on individual development and longevity by inducing ROS-mediated stress responses. For example, shortened lifespan and compromised tolerance to oxidative stress were observed when *Drosophila melanogaster* was exposed to sublethal doses of Ag nanoparticles 112. The life span of *Caenorhabditis elegans* was also reduced when the concentration of Ag nanoparticles exceeded 10 μg/mL 113. The clearance of NMNs is a big challenge. A certain number of NMNs were found in the liver, spleen, kidney, bone marrow, the central nervous system (CNS) and lymph nodes after inhalation, oral or intravenous injection 114, 115. The retention of NMNs in these tissues and organs is associated with metabolism, respiration, consciousness and immunity and can result in significant physiological and pathological effects, as shown in Figure 5. Human placenta is a multifunctional organ of particular interest because it controls material exchange between maternal and fetal tissues. The effect of NMNs on embryogenesis requires additional consideration. Increasing evidence has shown that transplacental transfer of nanomaterials also pose effects on embryogenesis 116. The results of *in situ* hybridization indicated that Ag nanoparticles caused a dose-dependent effect on embryonic development. Ag nanoparticles treatment led to delayed embryo development 117. Table 2 lists the summary of effects of NMNs on different tissues and organs.

### Effects of NMNs on metabolic-related tissues and organs

The gastrointestinal tract is in direct constant contact with the external environment. NMNs are found in various types of foods or food packaging materials 118. They can easily enter into human digestive system and be trapped in gastrointestinal tract 119. It is demonstrated that smaller nanomaterials pass through gastrointestinal tract more easily 120. After entering vascular circulation, NMNs can be efficiently excreted through the kidney along with the urine. Kidney is the core organ of metabolism and is involved in a range of crucial biological functions 121. As the most effective way of NMNs excretion, renal clearance of NMNs is favorable due to reduced possible toxic effects because it requires minimal interaction with the body 122. The emergence of renal clearable NMNs has made it possible toward addressing challenge of nonspecific accumulation of therapeutic NMNs 123. However, larger NMNs (larger than 6-8 nm) cannot be efficiently cleared via kidney. They may undergo biliary excretion and be eliminated via gastrointestinal tract 42. This process is governed by the liver, the metabolic center of the body 124. The liver converts molecules into waste products that can then be excreted through the kidney, representing the primary route for the remove of nanomaterials that cannot be directly cleared by the kidney 125.

Compared with other organs, the liver and the kidney, which are in charge of NMNs removal, receive and accumulate external nanomaterials at much higher volumes. As a model of NMNs, metallic Au nanoparticles have been widely reported in various studies to evaluate the biological effects of nanomaterials. They can be easily synthesized with different shapes (balls, rods, dots) and sizes. Au nanoparticles after intravenous administration are easily detected in liver and most of the administered dose can be retained in liver for a long time 126. A bio‐distribution study of Au nanoparticles in rats via intravenous injection showed rapid accumulation of Au nanoparticles in the liver 127. Even for intratracheal instillation of Au nanoparticles, the uptake and transport of ultrasmall Au nanoparticles resulted in accumulation within the liver 124. By using quantitative imaging based on laser ablation inductively coupled plasma mass spectrometry, *Elci et al.* demonstrated that surface charge determined suborgan distributions of intravenously injected Au nanoparticles in the kidney, liver and spleen. Positively charged nanoparticles accumulated extensively in the kidney, especially in the glomeruli 128. All these studies have shown that NMNs are capable of distributing from exposure site (e.g. blood, gut) to the secondary organs (liver, kidney). Since clearance of NMNs may occur over a timescale from several minutes to even a few months, internalized NMNs can persist within the body for a long time, trapped in the kidney, liver, and reticuloendothelial system, causing great effects on these metabolic-related organs.

By interacting with cells of sub-organs, NMNs may affect the function and histopathology of metabolic-related organs. Changes in epithelial cell microvilli as well as intestinal glands were observed in the study of *in vivo* effects of Ag nanoparticles. The loss of microvilli reduced the absorptive capacity of intestinal epithelium and weight loss in mice occurred after Ag nanoparticles treatment 129. A study evaluated biological effects of Pt nanoparticles less than 1nm in size. The research showed that Pt nanoparticles did not cause obvious side effects in the lung, spleen, and heart. However, histological analysis revealed necrosis of tubular epithelial cells and urinary casts in the kidney. The mice treated with Pt nanoparticles exhibited dose-dependent elevation of blood urea nitrogen. But these toxic effects were not observed in mice injected with 8nm Pt nanoparticles, suggesting the toxicity of Pt nanoparticles may be reduced by increasing nanoparticles size 130. In analyzing the existing results from the perspective of NMNs liver toxicity, it was found that NMNs did not induce significance damage to the liver after entry into the body. *Wallin et al.* studied the fate of Au nanoparticles in mice by tail vein injection. The mice were killed at different exposure times and any pathomorphological changes in the liver were observed 126.* Cho et al.* found PEG-coated 4- and 100-nm Au nanoparticles after intravenous administration in mice shared 67.1% and 50.9% of the significantly changed genes, respectively, whereas histological analysis of the liver tissues did not indicate any pathological changes in all treatment groups 131.

The accumulation of NMNs in metabolic-related organs can be seen as a protective mechanism of the body. The liver is the main detoxifying organ of the body. Liver storage may reduce systemic toxicity of NMNs. The fact showed that some intact NMNs were detected throughout the cytosol in a smaller size than originally administered. This was confirmed by the detection of low-molecular Au nanoparticles with different sizes in the liver 132. NMNs tend to be digested or metabolized biologically or chemically in liver before being neutralized and stored in the body in an attempt to reduce toxicity. The degradation process was mainly based on phagocytic activity of liver Kupffer cells. Au nanoparticles were identified in almost all Kupffer cells several hours after injection. Although Au nanoparticles were distributed in liver quickly, hepatocytes did not assimilate Au nanoparticles. Transmission electron microscopic analysis revealed that they accumulated inside vesicular structures of macrophages 126. The same result was described by other authors 133. Enzyme inside macrophage lysosomes would digest the accumulated NMNs, which somehow reduced systemic toxicity of NMNs. Furthermore, in the study of nanoparticles toxicity assessed on rat liver slices, although Au nanoparticles were rapidly intrahepatic collected, no overt signs of cytotoxicity were observed due to lactate dehydrogenase release, methylthiazolyldiphenyl tetrazolium bromide and glutathione level reduction 133. These morphological or functional changes increase the body's tolerance to NMNs.

### Alteration of tissues and organs involved in respiratory system

Inhalation is considered to be the most important route of NMNs exposure, especially in occupational settings 134. After inhalation of particle laden air, particles will enter into respiratory tract following airstream. Some inhaled particles tend to travel along the original path to reach deep into the lung, while other particles will be adsorbed on airway surface due to sudden directional change or airway bifurcation 135. Generally, large particles are likely to be deposited in nasopharyngeal region. Those particles that fail to be captured in the nasopharyngeal region will retain in tracheobronchial region and further removed by mucociliary clearance. However, the remaining ultrasmall NMNs (<100 nm) may penetrate deep into the lung, translocate beyond epithelial barrier into interstitium where they can escape the clearance mechanism and accumulate in the lung for a long time 136, 137. The lung is the first port of entry for inhaled nanomaterials into the body. Many types of NMNs-based products, such as sprays, are likely to be inhaled by consumers during respiration and accumulate in the lung. Moreover, NMNs exposures by other pathways were also found in the lung 42, 138. Since NMNs are nondegradable, they can stay in the lung for a long time once entering respiratory tract. The accumulated NMNs in the lung will enter into different compartments of the cell. Due to particle-cell interactions, the small-sized NMNs may induce detectable alternations in the lung. Importantly, internalized NMNs will be potentially further transported into systemic circulation, accumulate in other organs involved in respiratory system such as the heart, thereby inducing direct effects on these respiratory related organs.

The most commonly used method to evaluate the short-term or long-term effect of NMNs to the lung is by using intratracheal instillation. Many studies have reported the induction of pulmonary inflammation after NMNs exposure 139, 140. As a crucial indicator of inflammatory reaction, lactate dehydrogenase index is useful to indicate pneumonocyte change 141. For example, *Francesca et al.* tested lactate dehydrogenase and glucuronidase activities of Ag nanoparticles treated alveolar macrophages. Significant lactate dehydrogenase release and glucuronidase induction indicated pro-inflammatory effect of Ag nanoparticles to the lung. Although such obvious changes occurred at cellular level, there were no significant alternation in histology and pathology 142. The same conclusion was draw by* Hong et al.* In their repeated-dose toxicity study of Ag nanoparticles, no death was observed in any group. Yellow discolouration of the lungs was observed in a few rats, but the haematology, serum biochemical test and histopathological analysis between control and Ag treated groups revealed no statistically significant differences 143. For seeking of hazard ranking of nanomaterials, the acute lung, liver and systemic responses were assessed in C57BL/6N mice for three different nanomaterials. Exposure to low dose of Ag nanoparticles was also not accompanied by obvious pulmonary change or cytotoxicity 144. Pulmonary inflammation is initially a defense response to exotic nanomaterials. When NMNs are deposited in the lung, the body tries to recruit more macrophages through inflammatory reaction to remove nanomaterials. Most of NMNs are phagocytosed and digested by pulmonary macrophages 124. Quantitative analysis of both short and long Ag nanowires inside the lung showed a small but significant reduction of Ag nanowires lengths. For both Ag nanowires, there was lung inflammation at day 1, but disappeared at day 21 145. The lung tries to inactive and digests internalized NMNs. However, the durability of nondegradable NMNs in the lung might finally cause pulmonary fibrosis, which eventually affects respiratory function.

The small-sized NMNs may also cause alternations of other respiratory-related organs, among which the heart has attracted more attention than other organs. Comparison of toxicity of Ag, Au and Pt nanoparticles in developing zebrafish embryos revealed that both Ag and Pt nanoparticles induced a concentration dependent drop of heart rate, touch response and axis curvature. Ag nanoparticles even induced cardiac morphology change, while Au nanoparticles treatment did not cause any variation of zebrafish heart 104. The decrease in heart rate induced by NMNs was also reported by *Lee et al* 146. Cardiovascular system changes upon inhaled NMNs exposure can be explained by the following mechanisms. On one hand, the small size characteristics of NMNs allow them enter blood circulation by passing through the thin alveolar-capillary wall and influence cardiovascular system directly. On the other hand, long-term and amplified inflammation responses lead to overflow of inflammatory factors into the blood, which provides signals to the CNS to regulate heart activity. Furthermore, the deposited NMNs in the lung may stimulate alveolar sensory receptors to further active cardiovascular system 147. The autonomic regulation of nervous system activity will eventually manifest as alternation of cardiac function.

### Alteration of consciousness-related tissues and organs

The CNS is the most important part of controlling regular physiological functions of animals. As one of the tightest barriers in human body, the BBB protects the CNS from toxins and large, hydrophilic molecules diffusing into cerebrospinal fluid 148. In order to protect against invading substances or organisms, the BBB plays a key role in guarding the brain parenchyma and keeping a stable environment for normal neuron activities 149. NMNs exhibit extraordinary properties and are considered as superior carriers for achieving the delivery of a broad range of therapeutics into the brain which is well protected by the BBB 150. More and more studies have demonstrated that NMNs could cross the BBB and further access the CNS via several different routes 151, 152. NMNs can access to the blood or lymph nodes and translocate to the brain, or direct access to the brain from nasal cavity via nose-brain transport 153. After crossing the BBB, nanomaterials have a tendency to accumulate in specific regions of the brain including olfactory bulb, hippocampus, cerebral cortex, and striatum 154, 155. The accumulated NMNs in brain may gain access to neural cells, such as astrocytes, neurons, and microglia 156. The interactions between NMNs and those cells could greatly affect the structure, function, or chemistry of the CNS, followed by a variety of cognitive changes 157.

After entering the brain, NMNs can cause direct alteration of the structure and activity of the neural system or the follow-up effects. The effects of NMNs on CNS are various, including oxidative stress, cell apoptosis and autophagy, immune responses, and neuroinflammation. The most commonly reported are the extensive production of ROS, which leads to oxidative stress and inflammation 151. The brain oxygen consumption accounts for nearly a quarter of the whole body's oxygen consumption. Hence, the brain is more sensitive to hypoxic injury than other tissues. Upon NMNs treatment, cytokines release in the brain may cause neuroinflammation, and then neuronal damage, which will finally affect associated brain functions 153. Many commonly used NMNs have been reported as potentially neurotoxic materials 158. Researchers have analyzed changes in brain tissue and related brain functions after NMNs treatment, and histological alterations of the brain were observed. Deposition of NMNs in neural tissues, cerebral cortex or hippocampus can affect associated brain functions such as attention, memory, and judgement 159.

*Rahman et al.* found intraperitoneal injection of Ag nanoparticles in mice induced oxidative stress and altered gene expression in the brain 160. inflammation- and oxidative stress-related brain alterations in adult mice were also observed after intravenous administration of Ag nanoparticles 161. Since the morphology of the brain may change under long-term NMNs exposure, *El-Drieny et al.* studied the histology of adult rat brain after long-term Au nanoparticles treatment. Au nanoparticles mainly deposited in the neurons of cerebral cortex and hippocampus as well as the epithelium of choroid plexus 162. The study of influence of Ag nanoparticles on neurological development showed Ag nanoparticles altered neurological development of zebrafish and resulted in small heads 163. These changes may finally lead to alternations of brain functions. *Truong et al.* found Au nanoparticles treated zebrafish exhibited hypo-locomotor and abnormal behavioral activities 164. Another example showed invasion of different sized Au nanoparticles to brain hippocampus caused impairment of learning and memory in a size dependent manner, which was associated with hippocampus functional change 165. Adaptive change in the brain such as early inflammatory warn the body to protect it from more serious damage, but long-term stimulation will cause irreversible effects like morphological change in the brain. All these changes in consciousness-related tissues and organs allow us to better understand both beneficial and negative impacts of NMNs on the brain.

### Modulation of tissues and organs participating in immune response

The immune system is in charge of defending against foreign substances in order to protect the host. It is divided into innate immunity and adaptive immunity which are considered as two equally crucial aspects of the immune system. The innate immunity is nonspecific and the first line of defense of human body. It plays an essential role in early recognition of foreign substances and subsequent defense responses. Unlike innate immune immunity, the adaptive immune system is antigen-specific and reacts with organisms that induce the response 166. The innate immunity and adaptive immunity together form a sensitive target for xenobiotic induced toxicity. When exposed to the body, most NMNs are recognized as foreign antigens and thus elicit immune response. Although proteins in biological environment may adsorb to NMNs “foreign” surface and result in poor recognition by immune cells, however, most NMNs cannot escape immune recognition 167. Since innate defense system is the first in contact with foreign materials entering into the body, most studies of immunotoxicology induced by NMNs have focused on changes in innate immune system 168. There are enriched innate immune systems particularly in tissues that come into contact with external environment such as skin, respiratory mucosa, and gastrointestinal tract. However, NMNs may transport and penetrate through epithelial barriers to the blood due to ultrasmall size. Inside the body, NMNs will be endocytosed by immune cells, accumulate in tissues and organs of immune system, and interact with those organs 169. These direct interactions have profound effects on tissues and organs participating in immune response, which may be shown as activation or inhibition of immune function 170.

The most frequently mentioned immunological organ affected by NMNs is the spleen. The spleen is warehouse of the blood and is responsible for filtering the blood. As the largest lymphoid organ, the spleen is important both in innate and adaptive immune responses 171. NMNs can easily accumulate in the spleen after being phagocytosed, causing adaptive changes of the spleen both in morphology and function. The intravenously injected Ag nanoparticles with large particle size (80, 110 nm) had the highest concentration in the spleen compared to that of small particle size (20 nm). Larger NMNs are more likely to be recognized by macrophages than smaller sized one and are more likely to be trapped in the spleen 172. Moreover, particle size may influence their distribution in the spleen. Accumulation of Ag nanoparticles were found in the spleen, mesenteric lymph nodes, and axillary lymph nodes. 20 nm Ag nanoparticles was only distributed in the red pulp of the spleen, while 100 nm Ag nanoparticles is distributed in both the red and white pulp of the spleen 173. The widespread distribution of NMNs in the spleen is a manifestation of the body trying to remove foreign particles, which may cause morphology change of the spleen. Splenomegaly is often reported after nanoparticles deposition. In attempt to clear internalized NMNs, immune response will be activated to reduce toxicity. Lymphocyte proliferation is an important phase in immune response. It was shown in the research of *Wim* and co-workers that the number of T cells, B cells, and NK cells in the spleen increased sharply as well as the level of antibody IgM and IgE after Ag nanoparticles exposure, resulting in significant increase of spleen weight 173. This suggested that NMNs are able to regulate immune response by activating lymphocyte. Based on this conclusion, *Marc et al.* analyzed whether tumor presence would influence nanoparticles circulation since the immune system is altered when tumor exist. Due to an increase of M2-like macrophages, tumor presence significantly increased the clearance of nanoparticles from blood circulation, resulting in increased accumulation in the liver and spleen 174.

The immune response is an adaptive response involving multiple cell types and mediators. After entering human body, NMNs are recognized by “detectives” in the body and trigger a series of immune responses to try to get rid of them. Their relationship is like a hunter and a killer. The modulation of tissues and organs participating in immune system upon NMNs exposure enables the body react quickly and become adaptative to NMNs stimulus in a short time. However, this may be a double-edged sword. The adaptive changes induced by NMNs under a long period of time may cause pathological changes of immune-related tissues and organs, which lead to damage of immunological functions. The mice spleen showed obvious pathological changes under constant stimulation of Au nanoparticles, which were observed in form of distorted lymphoid architectures, minimized lymphoid follicles, diffused white pulp, and increased giant macrophages 175. Long term retention of NMNs in the spleen even lead to potential genetic changes. *Suresh et al.* examined gene expression in rat liver and spleen after intravenous administration of Au nanoparticles. Biodistribution of Au nanoparticles in the spleen at 1 day, 1 week, 1 month and 2 months were observed. The spleen showed significant changes in genes related to defensive reaction. Multiple genes involved in external stimuli, wound healing, and coagulation were downregulated, suggesting reduced spleen response external stimuli after long time Au nanoparticles treatment 127.

## Conclusion and perspectives

The ever-growing application of NMNs in therapeutics, diagnostics and bioimaging fields have yielded significant positive impacts toward the betterment of human health. The popularization of diverse NMNs-based products makes us more concern about their fate after entering human body. Inside the body, NMNs will distribute to specific tissues and organs, target cells and eventually interact with subcellular components. In order to better accommodate NMNs stimulation, cells and tissues will undergo various adaptive changes both *in vitro* and *in vivo* for better survival. Cellular and subcellular morphology changes are the first and most easily observed. Cells tend to become more rounded from stretched state to reduce their exposure of nanomaterials 185. The accumulation of NMNs in different subcellular components and their interaction with core organelles such as lysosomes, ER, Golgi apparatus, mitochondria, and nucleus will interfere their functions and cause influence on a variety of cellular functions. Cells try to adjust their digestion, protein synthesis and secretion, energy metabolism, mitochondrial respiration, and proliferation in response to intracellular foreign bodies. For example, the induction of autophagy is a defense and stress regulation mechanism of cells to try to make up the missed degradative function after lysosome swelling 70, 186. Changes in protein processing and cell adhesion are the result of cells to better withstand NMNs stimulation 76. Even under conditions where oxygen is sufficient, cells treated with NMNs might shift energy homeostasis as an adaptive process to satisfy energy demand for their survival 89. Moreover, adaptive changes of cells induced by long-term NMNs are different from that induced by short-term exposure, and long-term exposed cells increase their adaptability to NMNs stimulation 35, 43. When it comes to a cluster of cells, these cumulative effects are manifested as adaptive changes in tissues and organs. The chronic effects of NMNs on human body are of great interest since most MNNs are nondegradable and stay in tissues and organs for a long time. Accumulation of NMNs in metabolic-, respiratory-, consciousness- and immune-related tissues and organs seems to trigger significant physiological and pathological effects on core bodily functions. For instance, the liver is a detoxifying organ. Before being neutralized or stored in the body, NMNs tend to be digested or metabolized in the liver to reduce toxicity. When NMNs are deposited in the lung, pulmonary inflammation is initially a defense response to exotic nanomaterials. Changes in the brain such as early inflammatory warn the body to protect it from more serious damage. The body tries to recruit more macrophages through inflammatory reaction to remove nanomaterials. The immune system is in charge of defending against foreign substances in order to protect the host cells. For the body, to trigger a series of immune responses is a typical example to protect the body against NMNs exposure 167, 187. Definitely, under long-term stimulation of NMNs, there may be many adverse and even irreversible effects. Multi body functions will be affected, like energy supply, respiratory behavior, and even cognitive level. Premature senescence will appear under long-term stress induced by NMNs 98. When these adverse changes are beyond self-repair ability, cell death may occur via apoptosis or necrosis 104. The long-time retention of NMNs will have adverse effects on individual development and life span 112. Moreover, NNMs can also cross the placental barrier and pose effect on embryogenesis, which requires additional consideration 117.

Detailed investigation of interactions between NMNs and human body provides more comprehensive references for their applications. Ideally, these adaptive changes induced by NMNs can be exploited to create fantastic nanosystems. Through in-depth study of these adaptive changes, on one hand, we can take advantages of favorable adaptive changes, on the other hand, unfavorable changes can be avoided as much as possible to maximize the expected effects. The growing incidence of cancer is the biggest culprit of human death. At the cellular level, rapid tumor growth and proliferation depend on normal function of cancer cells, which is strongly associated with various roles of cellular and subcellular components. Thus, the adaptive changes followed by NMNs treatment can be designed to affect metabolism of cancer cells and enhance cancer cells killing. By affecting different cellular processes like cellular digestion, metabolism, respiration, proliferation as well as cell morphology, tailored NMNs provide great potential to increase sensitivity of cancer cells 10. For example, cellular morphology integrity is crucial for tumor invasion and metastasis 55. Alterations in cytoskeleton, organelles and other subcellular structures by NMNs treatment make cancer cells more susceptible to chemotherapy 188. Mitochondria respiration is directly associated with a variety of signal pathways that involved in apoptosis and necrosis. This highlights the usage of NMNs in drug resistant reversal through disruption of mitochondria energy metabolism 89. Also, NMN-induced autophagy, DNA dysfunction, ER stress and cell cycle redistribution have huge potential for smart therapeutic nanosystem design 189-191. In animal, the immune response initiated by NMNs as a protective mechanism can be well utilized with tumor immunotherapy to amplify immune efficacy 192. However, not all adaptive changes induced by NMNs are beneficial. For example, the accumulation of NMNs in detoxifying organs is seen as protective responses to reduce systemic toxicity of NMNs. But for therapeutic proposes, we hope that drug loaded NMNs circulate in the body for a long time and accumulate in specific lesions. Thus, various modifications of NMNs have been used to limit their side-effects. The size of nanomedicine is controlled to minimize their accumulation in metabolic organs and prolong their blood circulation time 193. The surface charge and shape of specific NMNs are also important to their *in vitro* and *in vivo* fate. For example, spherical NMNs are most commonly used drug carriers since renal clearable NMNs are generally spherical. Nanostructures with high aspect ratios but diameters smaller than kidney filtration threshold are also found to be renal clearable and therefore nonspecific accumulation in healthy tissues/organs is reduced 123. Surface charge affects *in vivo* fate of NMNs by influencing opsonization process. The density of surface charge impacts plasma protein adsorption both qualitatively and quantitatively, which, subsequently, has critical impacts on blood circulation and biodistribution of NMNs. High surface charge densities usually result in faster blood clearance while neutral charged NMNs contribute to extended blood circulation and reduced reticuloendothelial system clearance 194. Multiple factors control the circulation and organ clearance of NMNs. Structure design and surface modifications of NMNs are crucial to improve the biocompatibility, *in vivo* metabolism and adverse changes induced by NMNs. The size, shape, surface charge, and functional group of NMNs as well as tissue microenvironment could be monitored according to different purposes to reduce their side effect, change their blood circulation time, and regulate specific retention profiles within organs. Glutathione-mediated biotransformation is a well-known detoxification process in the liver to eliminate small xenobiotics. By designing a glutathione-coated Au nanoprobe that can bind to serum protein and be transported to the liver, *Jiang et al.* used detoxification process in the liver as a bridge toward maximizing targeting and minimizing nanotoxicity 195. Excessive immune response can damage the normal function of the body. The adaptive change of immune system under long-term NMNs stimulation may cause immunological function damage. Moreover, rapid identification by immune system results in rapid elimination of nanomedicine in human body. In order to avoid these problems, surface group modified and cell membrane coated NMNs are commonly used to escape immune recognition 196, 197. The investigation of interactions between NMNs and cells allows us to screen the most suitable NMNs to achieve maximum benefits, thereby taking advantage of favorable adaptive changes induced by NMNs and limiting their side-effects through surface modifications.

Here, an in-depth understanding of interactions among NMNs and cells, tissues, and organs are provided. Systematic study of adaptive changes induced by NMNs *in vitro* and *in vivo* enables us to better utilize NMNs for smart nanosystem design. Still, there is a lot of work to be done before a comprehensive understanding of NMNs-related adaptive changes is achieved. Apart from aforementioned observations, little is known about the fate of NMNs in human body. Detailed comparative studies on long-term and short-term effects of NMNs are lacking. Sometimes there is no obvious boundaries between adaptive changes and toxicities caused by NMNs. Most importantly, the choice of animal model is also a problem which needs to be considered carefully since many animal models we used now cannot fully reflect the complex physiological environment of human body. To elucidate the interplay of NMNs with human body, further in-depth research of *in vivo* tracking of NMNs is expected. Moreover, complete analysis of NMNs kinetics after administration needs to be conducted. We hope that the adaptive effects induced by NMNs will attract more and more interests for the design of smart nanopharmaceuticals, and it is possible that in the near future, advances in nanobiotechnology could enhance our capacity to make full use of these adaptive changes for improving the quality of human life.

## Figures and Tables

**Table 1:** Different types of NMNs and their main applications in academic studies.

Type of NMNs	Shape	Properties	Applications	References
Au nanoparticles	Nanostars	Large absorption of near-infrared light	PTT/ PDT agents for cancer treatment	11
	Nanoshell	Hyperthermia	Disrupt overactive sebaceous glands	12
	Spherical	Antibacterial	Inhibition of vancomycin-resistant enterococci, *E. coli* and* S. aureus*	13
	Spherical	Antiviral	Inhibition of HIV-1	14
	Spherical	SERS	*In vivo* multiplex molecular imaging	15
	Spherical	Osteoinductive	Osteoinductive agent for implant dentistry	16
Ag nanoparticles	Nanocrystalline	Anti-angiogenic	Combat multidrug-resistant cancer	17
	Spherical	Antibacterial	Prevention of tuberculosis	18
	Spherical	Antifungal	Inhibitory action against *Candida albicans, C. tropicalis*, *trichophyton mentaprophytes, C. glabrata* and* C. krusei*	19
	Crystalline	Antiprotozoal	Inhibitory action against *cryptosporidium parvum* in water purification	20
	Spherical	Sturdy and durable	Dental resin filler composite	21
Pt nanoparticles	Spherical	Strong affinity with dopamine	Dopamine sensors	22
	Spherical	Antioxidant	ROS scavenge	23
	Spherical, cubic, flower	Antibacterial	Inhibitory activity against *P. aeruginosa*	24
	Spherical	Electrocatalytic	Detection of hydrogen peroxide in cells	25
Pd nanoparticles	Spherical	Catalytic properties	Cancer treatment	26
	Sheet	LSPR/photothermal ablation	As PTT agents for cancer treatment	27
Ru nanoparticles	Spherical	Antibacterial	Inhibition of Gram-positive and Gram-negative bacteria	28
	Spherical	Promote osteogenic differentiation	Modulate the behavior of stem cells	29
Rh nanoparticles	Shell, frame, plate	Photothermal	Cancer phototherapy	30
Ir nanoparticles	Spherical	Photosensitive	Enhanced photodynamic performance	31
	Spherical	Hydrophobicity	Drug delivery	32
	Spherical	Charge transfer	Bioimaging	33

**Table 2:** Summary of the effects of NMNs on different tissues/organs.

Tissues and organs	Type of NMNs	Model	Physiological and pathological effects	References
Liver	Ag nanoparticles	Rats	Liver cytoplasmic vacuolization, no effect in hematology and biochemical parameters	176
	Au nanoparticles	Mice	Significant change in genes, but histological analysis did not show any pathological changes	131
	Au nanoparticles	Mice	No pathomorphological changes	126
	Au nanoparticles	Rats	Lactate dehydrogenase release and glucuronidase induction, pro-inflammatory effect	133
Kidney	Pt nanoparticles	Mice	Necrosis of tubular epithelial cells and urinary casts, dose-dependent elevation of blood urea nitrogen	130
	Ag nanoparticles	Rats	Dose-dependent effect on alkaline phosphatase and cholesterol. More accumulation in kidneys of female than male	177
	Au nanoparticles	Mice	Renal fibrosis	79
Stomach	Ag nanoparticles	3D-organoid models	Form complexes with *Helicobacter pylori*, attenuate *H. pylori* infection	178
Intestine	Ag nanoparticles	Mice	Loss of microvilli, reduced intestinal epithelial absorption and reduced weight	129
Spleen	Ag nanoparticles	Rats	Immune cells in the spleen increased sharply as well as the level of antibody, Spleen weight increased	173
	Au nanoparticles	Mice	Distorted lymphoid architecture, minimized lymphoid follicles, diffused white pulp	175
	Au nanoparticles	Rats	Significant change in genes related to defensive reaction	127
Lung	Au nanoparticles	Mice	Only star-like Au nanoparticles are able to accumulate in the lung, actual penetration into parenchyma	179
	Ag nanoparticles	Rats	Yellow discolouration of the lung, but no haematological and histopathological change	143
	Ag nanoparticles	Rats	Increases in alveolar inflammation and small granulomatous lesions	180
	Ag nanowires	Rats	Lung inflammation at day 1, but disappeared by day 21	145
Heart	Pt nanoparticles	Zebrafish	A concentration dependent drop of heart rate, touch response and axis curvature	104
	Ag nanoparticles	Zebrafish	Decrease in heart rate	146
Brain	Ag nanoparticles	Mice	Induce oxidative stress and altered gene expression	160
	Ag nanoparticles	Mice	Inflammation	161
	Au nanoparticles	Zebrafish	Hypo-locomotor activity and abnormal behavioral activity	164
	Au nanoparticles	Mice	Hippocampus functional change, impairment of learning and memory in a size dependent manner	157
Neuron	Ag nanoparticles	Zebrafish	Altered neurological development and result in small heads	163
Pancreas	Au nanoparticles	Diabetic rats	Increase relatively ROS factors	181
	Au nanoparticles	Mice	Inhibit matrix deposition, enhance angiogenesis	8
Reproduction	Ag nanoparticles	Rats	Altered testicular histology and sperm morphology abnormalities	182
	Ag nanoparticles	Mice	Atrophy of seminiferous tubules and absence of spermatids	183
	Ag nanoparticles	Mice	Microgranulomas in endometrium	184

## Abbreviations

Au: gold
Ag: silver
BBB: blood brain barrier
CNS: central nervous system
CT: computed tomography
ER: endoplasmic reticulum
Ir: iridium
LSPR: localized surface plasmon resonance
MMP: mitochondrial membrane potential
NMNs: noble-metal nanostructures
Os: osmium
Pd: palladium
Pt: platinum
PEG: polyethylene glycol
PTT: photothermal therapy
PDT: photodynamic therapy
ROS: reactive oxygen species
Rh: rhodium
Ru: ruthenium
SERS: surface-enhanced Raman spectroscopy
UPR: unfolded protein response
VEGF: vascular endothelial growth factor
